# Pathogenicity and Transmissibility of Goose-Origin H5N6 Avian Influenza Virus Clade 2.3.4.4h in Mammals

**DOI:** 10.3390/v14112454

**Published:** 2022-11-05

**Authors:** Cheng Zhang, Huan Cui, Ligong Chen, Wanzhe Yuan, Shishan Dong, Yunyi Kong, Zhendong Guo, Juxiang Liu

**Affiliations:** 1 College of Veterinary Medicine, Hebei Agricultural University, Baoding 071000, China; 2 Changchun Veterinary Research Institute, Chinese Academy of Agriculture Sciences, Changchun 130122, China

**Keywords:** H5N6, goose, chicken, pathogenicity, transmissibility

## Abstract

Throughout the last decade, H5N6 avian influenza viruses (AIVs) circulating in poultry and infecting humans have caused increasing global concerns that they might become a pandemic threat to global health. Since AIVs could occasionally cause asymptomatic infections in geese, virus monitoring in such a host should be critical to the control of cross-species infection. In addition, previous studies showed that clade 2.3.4.4h H5N6 AIVs could infect mammals without adaptation. However, the pathogenicity and transmissibility of goose-origin clade 2.3.4.4h H5N6 AIVs in mammals remain unknown. In this study, two H5N6 AIVs were isolated from a domestic chicken (A/chicken/Hebei CK05/2019 (H5N6)) and a goose (A/goose/Hebei/GD07/2019(H5N6)). This study is the first to evaluate the pathogenicity and transmissibility of goose-origin clade 2.3.4.4h H5N6 AIVs in mammals by comparison with chicken-origin 2.3.4.4h H5N6 AIVs. The CK05 virus had an affinity for α-2,3-receptors, while the GD07 virus had an affinity for both α-2,3-and α-2,6-receptors. The GD07 virus had a higher replication capacity in vitro and more severe pathogenicity in mice than the CK05 virus. The CK05 virus could not be transmitted effectively among guinea pigs, whereas the GD07 virus could be transmitted through direct contact among guinea pigs. The results of this study indicated the potential health threat of clade 2.3.4.4h H5N6 AIVs to mammals and emphasized the importance of continuous monitoring of H5N6 AIVs, especially in waterfowl.

## 1. Introduction

There are various host animals for avian influenza viruses (AIVs), such as wild or domestic birds and mammals [1,2]. Waterfowl are the natural reservoir host of AIVs and have made a major contribution to the spread of AIVs [3]. Some AIVs, including H5 and H7, are highly pathogenic avian influenza viruses (HPAIVs) that can cause severe disease in poultry and even pose a potential threat to human health [4].

In a previous study, two novel H5N6 AIVs were isolated in 2014–2015 from wild birds in southern China that appeared asymptomatic [5]. The phenomenon of asymptomatic infection in geese may become a blind spot for monitoring AIVs, which may lead to the stealthy spread of AIVs to the surrounding environment and animals without warning [4]. Therefore, the monitoring of AIVs in goose is a key link to control and evaluating transmission of influenza. The clade 2.3.4.4h AIVs became the dominant H5N6 lineage in China during 2018–2020 through continual evolution [6,7]. A study of swan-origin H5N6 AIV isolated in 2020 and a study of H5N6 AIV isolated from wild duck feces in 2022 both showed that the clade 2.3.4.4h H5N6 AIVs could infect mammals without adaptation and have certain pathogenicity [8,9]. Thus, the continuous detection of clade 2.3.4.4h H5N6 AIVs in geese is of significance to influenza epidemic prevention.

Mice are a widely used animal model to study the pathogenesis of AIVs [10], and guinea pigs are a good model for evaluating the transmissibility of AIVs [11]. This study is the first to evaluate the pathogenicity and transmissibility of goose-origin clade 2.3.4.4h H5N6 AIVs in mammals by comparison with chicken-origin 2.3.4.4h H5N6 AIVs. These results highlight the potential threat of goose-origin Clade 2.3.4.4h H5N6 AIVs to public health and livestock development.

## 2. Materials and Methods

### 2.1. Animal Ethics Statement

Experimental protocols involving animals were approved by the Animal Care and Use Committee of the Changchun Veterinary Research Institute, Chinese Academy of Agricultural Sciences (approval number: SCXK 202000599), and complied with regulatory and institutional guidelines. At the Changchun Veterinary Research Institute, all experiments with the influenza A (H5N6) virus were conducted in an animal biosecurity level 3 laboratory.

### 2.2. Viruses

A/chicken/Hebei/CK05/2019(H5N6) (abbreviated as CK05) (GenBank: MZ801736-MZ801738, OP601599- OP601603) was isolated from the cloacal swabs of domestic chickens with influenza-like symptoms in Hebei Province. A/goose/Hebei/GD07/2019(H5N6) (abbreviated as GD07) (GenBank: MZ817943-MZ817945, OP601604-OP601608) was isolated from cloacal swabs of healthy domestic geese in Hebei Province. All swabs were collected in 2 mL of phosphate-buffered saline (PBS). Then, the supernatant was filtered with 0.22 µm filters (Millipore). Then, the filtered liquid was inoculated into 9-day-old specific-pathogen-free (SPF) embryonated chicken eggs and incubated at 37 °C. After 48 h of incubation, allantoic fluids were harvested and stored at −80 °C.

### 2.3. Viral Genome Sequencing and Analysis

The QIAamp Viral RNA Mini Kit (Qiagen, Germantown, MD, USA) was used to extract viral genomic RNA from allantoic fluid according to the manufacturer’s instructions. The PrimeScript™ RT Reagent Kit with gDNA Eraser (TaKaRa, Dalian, China) was used to transcribe viral genomic RNA into cDNA. PCR amplification was performed using primers specific to AIV as previously reported [12]. PCR products were purified using the TaKaRa MiniBEST DNA Fragment Kit Ver.4.0 (TaKaRa, Dalian, China). A BigDye^™^ Terminator V3.1 cycle sequencing kit (Applied Biosystems, Foster City, CA, USA) was used for sequencing. The SEQMAN program was used to analyze sequencing data (DNASTAR, Madison, WI, USA). From NCBI GenBank, reference sequences for the HA, NA and PB2 genes were retrieved. With Cluster W, the downloaded sequences were aligned and compared to the strains used in this work. The MEGA 7.0.21 program (Sinauer Associates, Inc., Sunderland, MA, USA) was used to perform a phylogenetic analysis based on the maximum likelihood (ML) with a bootstrap value of 1000. Figtree (v1.4.2, http://tree.bio.ed.ac.uk/software/figtree/, accessed on 13 August 2022) was used to visualize the phylogenetic tree.

### 2.4. Receptor-Binding Assay

The receptor-binding assays were conducted as we described previously [13]. Briefly, HA assays were used to determine the receptor-binding preferences of the viruses using four different types of red blood cells (RBCs): chicken RBCs (cRBCs) containing α-2,3 and α-2,6-linked sialic acid (SA) receptors (Solarbio, Beijing, China, S9454); sheep RBCs (sRBCs) containing α-2,3-linked SA receptors (Solarbio, Beijing, China, TX0030); cRBCs treated with TaKaRa α-2,3-sialidase (TaKaRa, Dalian, China), which only left α-2,6-linked SA receptors; and cRBCs treated with Vibrio cholerae NA (VCNA; Roche), which left no receptors. The poultry isolate A/chicken/Hebei/HB777/2006 (H5N1) and human isolate A/California/04/2009 (H1N1) were used as controls for preferential binding to α-2,3-linked SA receptors and α-2,6-linked SA receptors, respectively. Next, 50 μL of the virus was added and serially diluted in PBS in 96-well plates. Finally, different 1% RBC suspensions of 50 μL were added. The titer was determined after 30 min of incubation at 25 °C.

### 2.5. Growth Kinetics of Viruses

Two H5N6 viruses were infected in triplicate into a Madin–Darby canine kidney (MDCK) or A549 human lung cancer cells at an MOI of 0.01, and the cells were then incubated at 37 °C in 6-well plates [14]. Cell supernatant was collected at 12, 24, 36, 48, and 60 h post-inoculation (hpi). The supernatant was then inoculated into 9-day-old SPF chicken embryos for a 50% egg infectious dose (EID_50_) determination to determine the viral titer of each sample collected.

### 2.6. Mouse Study

A mouse study was performed with reference to our previously published study [15,16]. Forty-eight six-week-old female BALB/c mice were purchased from Beijing Vital River Laboratory Animal Technology Co., Ltd. Fifteen BALB/c mice were randomly separated into three groups (*n* = five per group) and anesthetized with isoflurane. Two groups were inoculated intranasally with 50 μL of CK05 or GD07 at 10^6.0^ EID_50_. The control mice were inoculated intranasally with an equal volume of PBS. For a period of 14 days, the weight loss and survival rates of BALB/c mice in the three groups were observed daily. Thirty-three BALB/c mice were randomly separated into three groups (three for control and fifteen per group for inoculation). The animals in the two inoculated groups were anesthetized with isoflurane and intranasally inoculated with CK05 or GD07 virus at 10^6.0^ EID_50_, while the mice in the control group were intranasally inoculated with an equal volume of PBS. Three mice per inoculated group were euthanized at 1, 3, 5, and 7-days post-infection (dpi) for viral load in the lungs, heart, liver, spleen, kidneys, and brain. The six tissue samples were homogenized in 1 mL of PBS using a tissue lyser (Qiagen, Germany). Samples were centrifuged for 10 min at 8000 rpm at 4 °C. After centrifugation, the six tissue homogenates were inoculated into SPF chicken eggs and the EID_50_ was determined by hemadsorption. At 5 dpi, the lungs of BALB/c mice from the three groups were removed and fixed in formalin, embedded in paraffin, and stained with hematoxylin and eosin (H&E) for pathological examination. The 50% mouse lethal dose (MLD_50_) of the two strains was determined by inoculating groups of five BALB/c mice with 10-fold serial dilutions containing 10^1.0^–10^6.0^ EID_50_ of virus in a volume of 50 µL (total of 60 BALB/c mice). The results were calculated by using the method of Reed and Muench [17].

### 2.7. Guinea Pig Study

Three guinea pigs in each group were inoculated intranasally with CK05 or GD07 at 10^6.0^ EID_50_ at 200 μL. The next day, three uninfected and three infected guinea pigs were placed on the same side of the cage to facilitate direct contact transmission. Three uninfected guinea pigs in each group were placed on the other side of the cage with infected guinea pigs at a contact distance of 5 cm for aerosol transmission. Nasal washes were collected every two days. Viral titers were confirmed by titration on embryonated SPF eggs. Sera were collected to determine seroconversion at 21 dpi.

### 2.8. Statistical Analysis

Prism (GraphPad Software 8.0, San Diego, CA, USA) was used to determine statistical significance, using a one-way analysis of variance (ANOVA) (*p* < 0.05, *; *p* < 0.01, **; *p* < 0.001, ***). All assays were performed in at least three independent experiments. The error bars represent the standard deviation.

## 3. Results

### 3.1. Molecular Phylogenetic Analysis

**Figure 1:**
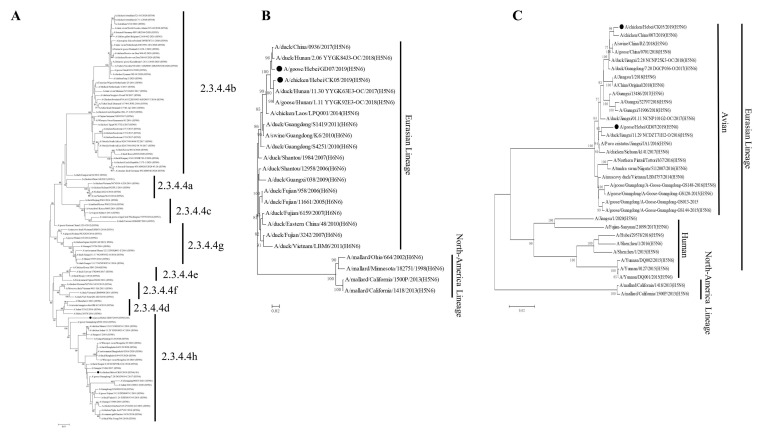
Phylogenetic trees of the HA, NA, and PB2 genes of the H5N6 (CK05 and GD07) viruses. (**A**) Phylogenetic tree of HA. (**B**) Phylogenetic trees of NA. (**C**) Phylogenetic trees of PB2. Sequences in this study are marked with black chicken or goose.

Homology analysis showed that the HA and NA genes of CK05 shared 96.0% and 97.4% nucleotide sequence identity, respectively, with the HA and NA genes of GD07. The two H5N6 AIVs isolates both shared the same amino acid sequence RERRRKR↓G on the HA proteins, indicating that they are HPAIVs [18]. In Supplementary Table S1, the amino acid differences of the two strains of virus in this study are shown. Moreover, the stalk domain of the NA protein in two strains has 11 amino acid deletions in the NA stalk region, implying that the viruses showed some adaptation and pathogenicity in mammals [19]. The S128P mutation was observed in the HA protein of GD07 virus, suggesting that the receptor specificity may be altered to increase the virus ability to infect mammals [20]. The mutation at T339K detected in the PB2 protein of the GD07 virus confers increased the pathogenicity of the H5N6 virus in mammals [21]. To examine genetic relationships using sequences obtained in this study and available sequences in the GenBank database, we constructed phylogenetic trees based on the HA, NA, and PB2 genes. The HA genes of the two H5N6 AIVs were found to be clustered in the 2.3.4.4h clade, according to the phylogenetic analysis (Figure 1A). The phylogenetic analysis of the NA and PB2 genes showed that both strains of H5N6 viruses were from the Eurasian lineage (Figure 1B,C). Moreover, the NA genes of the two H5N6 AIVs in this study were closely related to the NA genes of H6N6 viruses, and the PB2 genes were closely related to those of the avian isolates.

### 3.2. Receptor-Binding Preference

**Figure 2:**
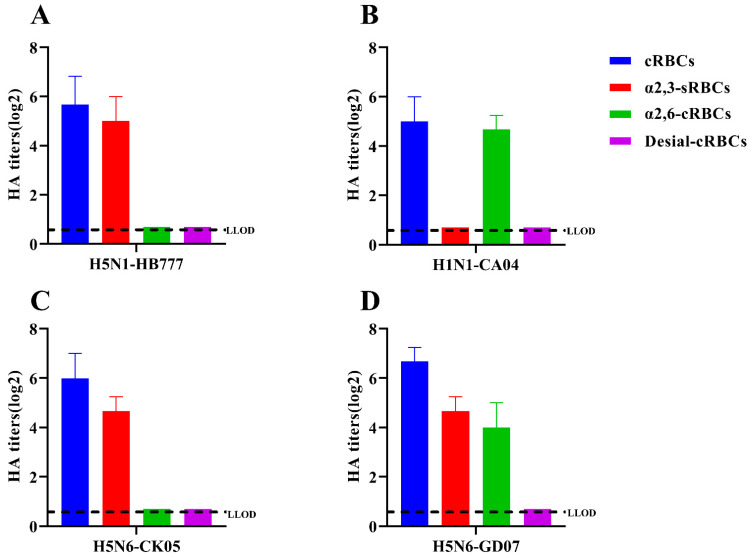
Receptor-binding specificity of H5N6 (CK05 and GD07) viruses. The receptor-binding specificity of viruses is determined by RBCs containing different SA receptors. Only avian-like (α-2,3) receptors were found in the control group, HB777(H5N1) (**A**), while only human-like (α-2,6) receptors were found in CA04(H1N1) (**B**). Avian-like (-2,3) receptors were found in both CK05 (**C**) and GD07(D). Human-like (-2,6) receptors are present only in the GD07 (**D**) strain. In each group, three separate experiments were carried out. The lower limit of detection (LLOD) is indicated with a black dotted line.

Sialic acid binding of the two H5N6 AIVs was characterized by a preference for RBCs, and the RBCs treated by 2,3-sialidase or 2,6-sialidase or by both 2,3-sialidase and 2,6-sialidase. A/chicken/Hebei/HB777/2006 (H5N1) α-2,3 connections were agglutinated with saliva acid receptor sRBCs, and A/California/04/2009 (H1N1) could agglutinate only α-2,3-sialidase-treated cRBCs containing α-2,6-linked sialic acid receptors. CK05, in this study, could only agglutinate sRBCs containing α-2,3-linked sialic acid receptors; GD07 could agglutinate not only sRBCs containing α-2, 3-linked sialic acid receptors but also cRBCs treated with α-2,3-sialidase, which contain only α-2, 6-linked sialic acid receptors (Figure 2). Compared to CK05, GD07 showed a similar binding preference to α-2,3 and α-2,6-linked sialic acid receptors, suggesting a potentially high risk for mammals.

### 3.3. Growth Kinetics of Viruses

**Figure 3:**
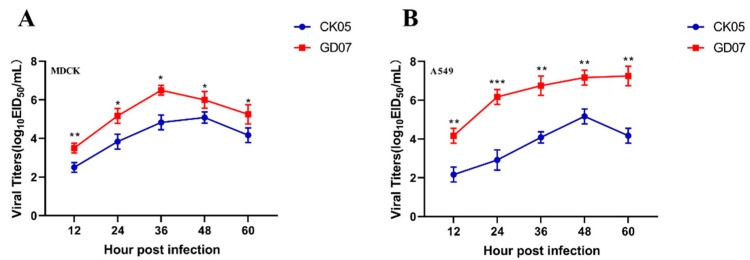
Viral titers at different time points in MDCK (**A**) or A549 (**B**) cells. The two strains (CK05 and GD07) infected cells with an MOI of 0.01 (10^5^ cells). At 12, 24, 36, 48, and 60 hpi, cell supernatants were collected and inoculated into SPF chicken embryos. The titer of the virus at different time points was determined by EID_50_. Three independent experiments were performed in each group. * *p* < 0.05, ** *p* < 0.01, *** *p* < 0.001.

Both MDCK cells and human A549 cells were used to study the replication kinetics of CK05 and GD07 (Figure 3). Figure 3A shows the replication ability of the virus in the MDCK cell lines. The viral titer of CK05 virus reached a peak at 48 hpi, and the titer was 10^4.8^ EID_50_/mL. The viral titer of the GD07 virus was highest at 36 hpi, 10^6.5^ EID_50_/mL. Figure 3B shows the replication ability of the virus in the A549 cell lines. The viral titer of the CK05 virus was highest at 48 hpi, 10^4.8^ EID_50_/mL. The viral titer of GD07 reached a peak at 60 hpi and the titer was 10^7.3^ EID_50_/mL. The GD07 viral titer in both cell lines was significantly higher than the CK05 viral titer at all collection times (*p* < 0.05) (Figure 3). Therefore, the in vitro replication capacity of the GD07 virus was significantly higher than that of the CK05 virus.

### 3.4. Pathogenicity in Mice

**Figure 4:**
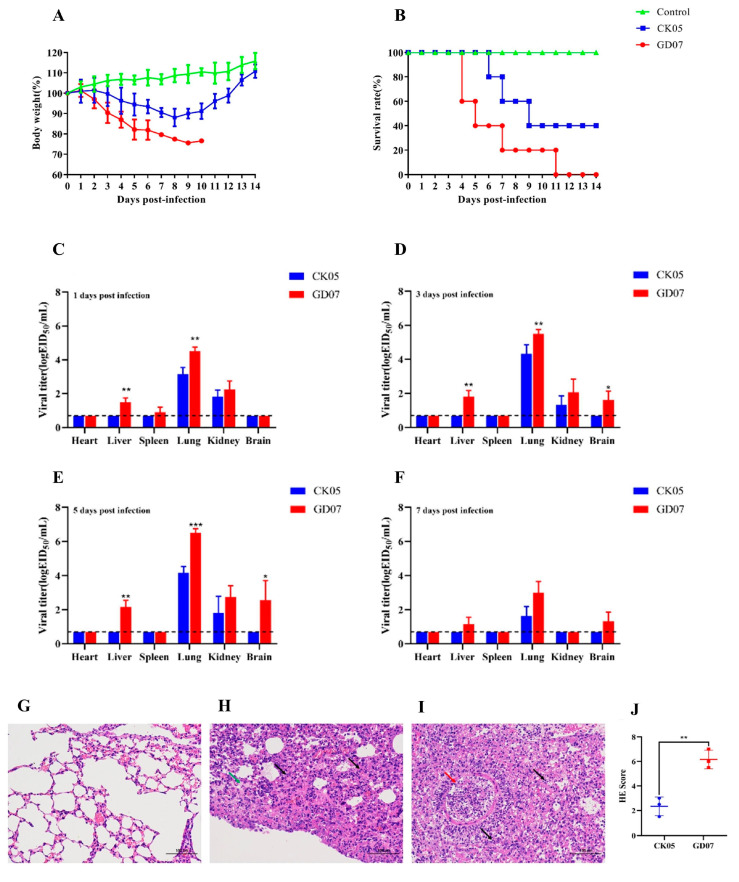
Pathogenicity of the isolated viruses in mice. All mice were intranasally inoculated with the H5N6 viruses at 10^6^ EID_50_. (**A**) The daily weight of each group was monitored for 14 days. (**B**) The survival rate of each group was recorded for 14 days. (**C**) The tissue distribution of the CK05 and GD07 virus in BALB/c mice at 1 dpi. (**D**) The tissue distribution of the CK05 and GD07 virus in BALB/c mice at 3 dpi. (**E**) The tissue distribution of the CK05 and GD07 virus in BALB/c mice at 5 dpi. (**F**) The tissue distribution of the CK05 and GD07 virus in BALB/c mice at 7 dpi. (**G**) Lung pathological sections of the BALB/c mice in the control group at 5 dpi. (**H**) Lung pathological sections of the BALB/c mice in the CK05 group at 5 dpi. (**I**) Lung pathological sections of the BALB/c mice in the GD07 group at 5 dpi. (**J**) Pathological scores in the lungs of infected BALB/c mice. Images were acquired using a ×20 magnification objective. Alveolar wall thickening, lymphocyte infiltration (arrow black); acidophilic protein-like exudation (arrow green); epithelial cell necrosis (arrow red). * *p* < 0.05, ** *p* < 0.01, *** *p* < 0.001. LLOD for viral titers is indicated with a black dotted line.

Figure 4 shows the pathogenicity of the two strains in mice. The weight of the mice in both groups decreased gradually after the challenge (Figure 4A). The CK05 group reached its lowest weight at 8 dpi, with body weight dropping to approximately 83%, and then, weight began to rise. In the GD07 group, body weight reached its lowest point at 9 dpi, dropping to approximately 76%. As shown in Figure 4B, the CK05 group began to die at 6, 7, and 9 dpi, with a survival rate of 40%. The GD07 group began to die at 4, 5, 7, and 11 dpi, and all died at 11 dpi. At 1 dpi, the virus was detected in the lung and kidney of the CK05 group and the liver, lung, and kidney of the GD07 group. At 3 and 5 dpi, the virus was detected in the lung and kidney of the CK05 group and the liver, lung, kidney, and brain of the GD07 group. At 7 dpi, only the lungs of the mice in the CK05 group and the liver, lung, and brain of the animals in the GD07 group were positive for the virus (Figure 4C–F). At 1, 3, and 5 dpi, the viral titer of the lung in the GD07 group was significantly higher than that in the CK05 group (*p* < 0.01), with the virus content highest at 5 dpi. As shown in Figure 4G–J, the mice infected with CK05 and GD07 showed significant lung damage. Pathological results were scored in each part of each lung: 0—no pathological changes; 1—lesion area ≤10%; 2—lesion area 10%–50%; 3—affected area ≥50%. When histological examination showed pulmonary edema and/or alveolar hemorrhage, the score was increased by 1 point. In comparison to that of the CK05 group, the pathological lung damage in the GD07 group was considerably higher (*p* < 0.01). The MLD_50s_ of the CK05 and GD07 viruses were 10^3.3^ and 10^5.3^ EID_50_, respectively. Data from all the mouse experiments indicated that the pathogenicity of the GD07 virus in mice was higher than that of the CK05 virus.

### 3.5. Evaluation of Transmission Capacity among Guinea Pigs

**Figure 5:**
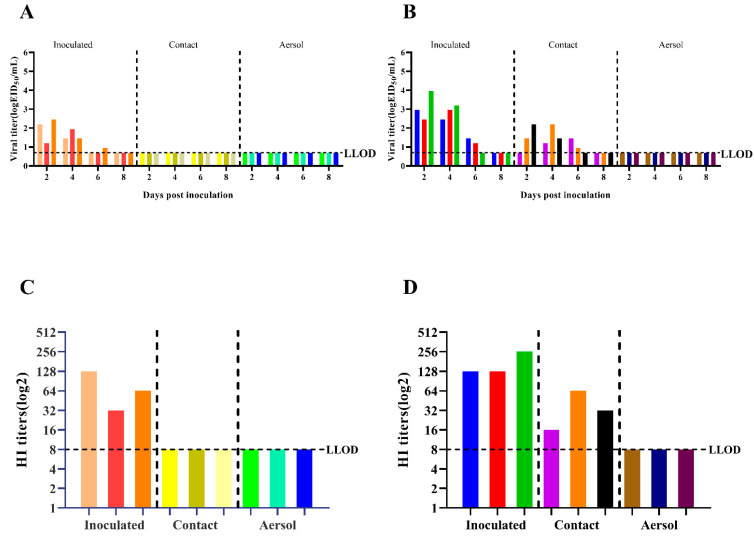
Transmission of H5N6 (CK05 and GD07) viruses in guinea pigs. (**A**) *X*-axis: guinea pigs in the groups infected with CK05, exposed to direct contact, and transmitted by aerosols. *Y*-axis: influenza virus titers in guinea pig nasal washes. (**B**) *X*-axis: guinea pigs in the groups infected with GD07, exposed to direct contact, and transmitted by aerosols. *Y*-axis: influenza virus titers in guinea pig nasal washes. (**C**) *X*-axis: guinea pigs in the CK05 infection group, direct contact group, and aerosol transmission group. *Y*-axis: HI antibody titers of different guinea pigs. (**D**) *X*-axis: guinea pigs infected with GD07 group, directly exposed group and aerosol transmitted group. *Y*-axis: HI antibody titers of different guinea pigs. Each color bar represents an individual guinea pig. LLOD is indicated with a black dotted line.

Figure 5A shows that the CK05 virus was not detected in the nasal wash of either the direct contact group or the aerosol-transmitted group. Figure 5B shows that at 4 dpi, the virus was detected in the nasal wash of three guinea pigs in the direct contact group on GD07, with a transmission efficiency of 100%. The virus was not detected in the nasal wash of the aerosol transmission group. These results indicate that only the GD07 virus can be transmitted through direct contact with 100% efficiency. The HI antibody titers of guinea pig serum samples are shown in Figure 5C,D. The serum of the infected group was positive, the serum of the aerosol transmission group was negative, the serum of the direct contact group was negative for the CK05 virus, and the serum of the direct contact group was positive for the GD07 virus.

## 4. Discussion

In this study, the GD07 virus exhibited enhanced pathogenicity in mice. The MLD_50_ of the GD07 strain was 10^3.3^ EID_50_, which was 100 times lower than that of CK05 (10^5.3^ EID_50_) and 200-1200 times lower than that of the clade 2.3.4.4h H5N6 strain previously isolated from waterfowl. The MLD_50_ of WS/XJ/1/2020(H5N6) and A/duck/Khunt lake/#500/2019(H5N6) in mice was 10^6.38^ EID_50_ and 10^5.68^ EID_50_, respectively [8,9]. Given the rapid spread of the H5N6 AIVs in avian and the ability to generate new recombinant strains, especially in waterfowl, increasing attention should be given to prevent the reassortment of novel AIVs to infect humans or other animal species.

According to phylogenetic analysis of the HA gene, the two H5N6 strains identified in this study are members of the clade 2.3.4.4h H5N6 AIVs, which is in concordance with the clade of H5N6 strains that predominated in China between 2018 and 2020, as described in earlier studies [22]. The two strains in this study had multiple basic amino acids at the cleavage site (RERRRKR↓G), which is a feature of HPAIVs [9,23,24]. Receptor binding preference is one of the important factors that affects the cross-species transmissibility of AIVs [25]. As shown in Figure 2, CK05 preferentially bound to avian-like (α-2,3) receptors, but GD07 had binding preferentially to both avian-like (α-2,3) receptors and human-like (α-2,6) receptors, which suggested that GD07 might potentially infect mammals. In previous studies, the alteration of receptor binding preference for H5N6 AIVs was found to be caused by HA S128P [20]. For the GD07 virus with the HA S128P mutation, this binding property of human-like receptors may make it possible to infect mammals [26,27,28]. The RNA-dependent RNA polymerase from influenza virus, which is composed of PA, PB1, and PB2 subunits, may be responsible for viral transcription and replication [29]. Sequence analysis revealed an amino acid substitution, PB2 T339K, in the GD07 virus. In previous studies, the amino acid 339 was found to be located in the cap-binding pocket of the H5N1 PB2 cap [21,30]. Functional investigations showed that the ribonucleoprotein complex with the K339T substitution could decrease RNA synthesis and influenza polymerase activity, and a reconstituted H5N1 virus with the K339T alteration had less pathogenicity in mice [21]. Figure 3 and Figure 4 demonstrate that the GD07 virus replicated more effectively than the CK05 virus in MDCK and A549 cells and caused more severe pathological damage in mice. These findings further demonstrate the importance of PB2 T339K in the replication and virulence of AIVs in mammals [31].

Most importantly, the contact transmission capability of the GD07 virus (100%) was higher than that of the CK05 virus (0%) (Figure 5), suggesting that HA S128P and PB2 T339K had the potential to overcome the species barrier between avian and mammals. Moreover, the joint contribution of HA and PB2 genes was shown to change virus virulence and transmissibility [32,33]. This adaptation to mammals might be caused by the multispecies culture environment in farms, where AIVs could be adapted and selected by continuous pressure from the complex surroundings of birds and mammals [34]. A previous study showed that the receptor binding ability might affect the spread of influenza virus [35,36,37], so we hypothesized that the direct contact transmissibility of GD07 might be caused by its receptor binding ability. Although this study did not use reverse genetic technology to verify our speculation, this issue needs to be further explored based on multiple amino acid analysis in the future.

## 5. Conclusions

In conclusion, these results indicated the potential health threat of clade 2.3.4.4h H5N6 AIVs to mammals and emphasized the importance of continuous monitoring of H5N6 AIVs, especially in waterfowl.

## Data Availability

The data is available upon request.

## Supplementary Materials

The following supporting information can be downloaded at: https://www.mdpi.com/article/10.3390/v14112454/s1, Table S1: Amino acid differences between two H5N6 influenza viruses.
