# Supplementary material for: Pathogenicity and Transmissibility of Goose-Origin H5N6 Avian Influenza Virus Clade 2.3.4.4h in Mammals
Source: Viruses. 2022 Nov 5;14(11):2454. doi: 10.3390/v14112454 (PMC9699601; doi:10.3390/v14112454)
Supplement: Supplementary file 1 [file viruses-14-02454-s001.zip › viruses-2004760-supplementary.pdf]

Supplementary Table 1. Amino acid differences between two H5N6 influenza viruses.

Segment	Position	CK05	GD07
HA	44	R	G
	59	D	N
	77	S	R
	120	Q	L
	125	R	S
	128	S	P
	146	A	P
	174	K	R
	188	S	N
	203	V	I
	288	R	S
	274	I	M
	325	S	N
	372	R	K
	400	V	I
	484	E	G
	532	V	A
	537	V	M
NA	80	N	S
	83	K	R
	228	H	Q
	267	A	T
	287	R	G
	304	V	I
	306	I	T
PB2	62	R	K
	112	P	T
	137	S	N
	144	R	K
	339	T	K
	384	V	L
	457	I	V
	473	M	I
	495	V	A
	524	T	M
	559	N	S
	613	A	V
	636	M	L
	699	R	K
	755	R	G
M	34	T	A

	183	T	A
	235	Y	C
	333	S	N
PA	216	K	T
	359	G	E
	546	V	A
PB1	22	V	A
	62	E	K
